# Variability of the Pharyngeal Phase of Swallow in the Cat

**DOI:** 10.1371/journal.pone.0106121

**Published:** 2014-08-29

**Authors:** Daniel G. Spearman, Ivan Poliacek, Melanie J. Rose, Donald C. Bolser, Teresa Pitts

**Affiliations:** 1 Department of Physiological Sciences, University of Florida, Gainesville, Florida, United States of America; 2 Institute of Medical Biophysics, Jessenius Faculty of Medicine, Comenius University, Martin, Slovak Republic; RWTH Aachen, Germany

## Abstract

**Objective:**

The pharyngeal phase of swallow has been thought to be a stereotypical motor behavior.

**Study Design:**

This is a prospective, preclinical, hypothesis driven, one group by three-task design.

**Methods:**

We sought to compare the effects of pharyngeal swabbing, water only, and water plus punctate mechanical stimulation on the spatiotemporal features of the pharyngeal phase of swallow in the cat. Swallow was elicited under these three conditions in six anaesthetized cats. Electromyographic activity was recorded from seven muscles used to evaluate swallow: mylohyoid, geniohyoid, thyrohyoid, thyroarytenoid, thyropharyngeus, cricopharyngeus, and parasternal.

**Results:**

Pharyngeal swabbing in comparison to the other stimulus conditions, results in decreases in post-swallow cricopharyngeus activity (upper esophageal sphincter); a significant increase in parasternal (schluckatmung; swallow breath) activity; and increases in thyrohyoid (laryngeal elevator), thyroarytenoid (laryngeal adductor) and parasternal muscles burst duration. Pearson correlations were found of moderate strength between 19% of burst duration comparisons and weak to moderate relationships between 29% of burst amplitude comparisons. However, there were no positive significant relationships between phase durations and electromyogram amplitudes between any of the muscles studied during swallow.

**Conclusions:**

The results support the concept that a stereotypical behavior, such as pharyngeal swallowing in animal models, can be modified by sensory feedback from pharyngeal mucosal mechanoreceptors. Furthermore, differences in swallow phase durations and amplitudes provide evidence that separate regulatory mechanisms exist which regulate spatial and temporal aspects of the behavior.

## Introduction

Swallowing consists of three phases: oral, pharyngeal, and esophageal, each of which participates in a bolus transport from the oral cavity to the stomach [1]–[7]. The pharyngeal phase of swallowing is the movement of a bolus from the pharynx to the esophagus [3], [8]–[10]. It can be initiated by touch, pressure, and/or similar action of a liquid on the tongue, faucial pillars, soft palate, uvula, epiglottis, pharyngeal wall, and/or junction of the pharynx/esophagus [5], [10], [11]. The pharyngeal phase is a reflexive patterned behavior that has been hypothesized as stereotypic [1], [2], [12]–[15]. Doty and Bosma [2] found no measurable difference in duration or amplitude of the measured muscles when swallow was elicited by superior laryngeal nerve stimulation, pharyngeal stimulation with a cotton swab, or rapidly injecting water into the pharynx. However, as noted by Thexton et al [15], [16], Doty and Bosma [2] observed considerable variation in electromyogram patterns of some upper airway muscles during swallowing. These investigators also observed non-swallow behaviors in their recordings, in particular, the aspiration reflex. This knowledge, along with a study by Patterson [17], motivated Thexton et al [15] to re-address some issues that Doty and Bosma [2] had studied. Thexton and coworkers [15], [16] and Sumi [18] confirmed pharyngeal muscle variation during swallowing in infant animals. These studies are consistent evidence in humans showing that the pharyngeal phase of swallow can be modified by bolus type, size, consistency, temperature, and taste i.e [9], [11], [19]–[28]. Additionally, multiple sensory modalities can influence swallow including: perceptual factors of food appearance, taste, and mechanosensory feedback from oral mucosa/tongue regarding bolus consistency potentially accounting for the aforementioned observations.

Considering the motor pattern variance, in human or awake-animal models, it is difficult to understand the role of feedback from oropharyngeal airway regions on the swallow motor pattern from previous work. In particular, separation of volitional factors as well as oral sensory feedback would reveal the influence of sensory feedback from sub-oral (pharyngeal) airway in modulation of the swallow motor pattern. For example, Patterson [17] in an anesthetized opossum model demonstrated significant differences in mylohyoid activation elicited by unilateral and/or bilateral superior laryngeal nerve stimulation compared to solely mechanical stimuli. However, electrical stimulation of the superior laryngeal nerve is a non-natural stimulus that induces swallowing through fixed frequency feedback to the brainstem. An additional complicating factor that makes it difficult to interpret the results of previous work in this area is the reliance on measuring the frequency of occurrence of swallow as the sole outcome. This approach does not acknowledge the complexities in motor drive to various muscles that produce this behavior. Knowledge of the spatiotemporal features of specific musculature activation is important in understanding the functional organization of central control systems that generate complex behaviors [29]–[32].

We have investigated this issue in an anesthetized, unparalyzed, cat model in which swallow is readily produced by water as well as purely mechanical stimuli applied to the pharynx with virtually no involvement of oral afferents. This model also benefits from a high degree of knowledge of the brainstem control system for behaviors involving respiratory muscles [29], [33]–[37] We hypothesized that spatiotemporal control of pharyngeal muscles involved in hyoid elevation and pharyngeal constriction would be modifiable based on stimulus modality.

## Methods

Experiments were performed on six spontaneously breathing adult male cats. The protocol was approved by the University of Florida Intuitional Animal Care and Use Committee (IACUC). The animals were initially anesthetized with sodium pentobarbital (Lundbeck, Inc., Deerfield, IL) (35 mg/kg i.v.); supplementary doses were given as needed. The right femoral artery and vein were cannulated to monitor blood pressure and administer i.v. fluids, respectively. Physiologic levels of end-tidal CO_2_ (4–4.5%) body temperature, and arterial blood gas composition were continually maintained and monitored.

Electromyograms were recorded using bipolar insulated fine wire electrodes according to the technique of Basmajian and Stecko [38]. Seven muscles were used to evaluate swallow function: mylohyoid, geniohyoid, thyrohyoid, thyropharyngeus, thyroarytenoid, cricopharyngeus, parasternal, and rectus abdominis. These muscles span the actions during the pharyngeal phase of swallow: a) mylohyoid, geniohyoid and thyrohyoid for hyolaryngeal elevation, b) thyropharyngeus for inferior pharyngeal constrictor, c) cricopharyngeus for upper esophageal sphincter, d) thyroarytenoid for laryngeal adduction, e) parasternal for inspiratory activity, and f) rectus abdominis for expiratory activity.

The digastric muscles were dissected away from the surface of the mylohyoid and electrodes were placed in the left mylohyoid. A small horizontal incision was made at the rostral end of the right mylohyoid followed by an incision down the midline for approximately 5 mm to reveal the geniohyoid muscle. Electrodes were placed 1cm from the caudal insertion of the geniohyoid muscle. The thyroarytenoid muscle electrodes were inserted through the cricothyroid window into the anterior portion of the vocal folds, which were visually inspected post-mortem. Minor rotation of the larynx and pharynx counterclockwise revealed the superior laryngeal nerve, which facilitated placement of the thyropharyngeus muscle electrodes. The thyropharyngeus is a fan shaped muscle with the smallest portion attached to the thyroid cartilage; electrodes were placed in the ventral, caudal portion of the muscle overlaying thyroid cartilage within 5 mm of the rostral insertion of the muscle. To place electrodes within the cricopharyngeus muscle, the larynx and pharynx were rotated counterclockwise to reveal the posterior aspect of the larynx. The edge of the cricoid cartilage was located by palpation and electrodes were placed in the cricopharyngeus muscle just cranial to the edge of this structure. Thyrohyoid muscle electrodes were inserted approximately 5 mm rostral to the attachment to the thyroid cartilage; those for the parasternal muscle were placed in the third intercostal space, just adjacent to the sternum. The positions of all electrodes were confirmed by visual inspection (following electrode placement and post-mortem) and by electromyogram activity patterns during breathing and swallow.

Swallowing was defined as a quiescence of cricopharyngeus (upper esophageal sphincter) activity with overlapping mylohyoid, geniohyoid, thyropharyngeus, thyrohyoid, thyroarytenoid and parasternal (schluckatmung or swallow breath) activity [39]–[42]. Swallow can be clearly differentiated from other behaviors (augmented breath, laryngeal elevation, cough, expiration reflex, and aspiration reflex) by this definition [15], [16], [43]–[45].

### Stimuli conditions

The stimulation protocol started approximately 1–2 hours following electromyogram placement. Three sensory stimuli of the pharynx were used to elicit swallows: water only, water plus punctate mechanical stimulation, and pharyngeal swabbing. To initiate water only swallowing, a one-inch long, thin polyethylene catheter (diameter 0.5–1.0 mm), attached to a 6 ml syringe was placed into the oropharynx. Water was injected into the pharynx via a syringe (3 ccs). Water plus punctate stimulation of the pharynx was conducted in the same manner except that the syringe was allowed to touch the oral/pharyngeal cavity simultaneously with water injection. During pharyngeal swabbing, the pharyngeal mucosa was mechanically stimulated through rotation of a cotton swab. The water injection, pharyngeal swabbing and punctate stimulus were directed at the same anatomical location, the junction of the oral cavity with the pharyngeal cavity including the posterior pharyngeal wall. Each condition was completed three times in sequence, and there was at least one-minute duration between each stimulation trial.

### Data processing and statistical analysis

Signals were recorded using sampling frequency of Hz. “Spike 2” Version 7 (Cambridge Electronic Design, United Kingdom) was used to automate the analysis process. Electromyograms were rectified and moving averages with the time constant of 50 ms were obtained. For duration measures a mean of the peak electromyogram signal for one- second preceding the swallow was set as a threshold marker. The duration measures were marked as *onset* when the signal was greater than the threshold level and *completion* when the signal was again less than or equal to the threshold level. The only exception to this was the metric of duration of relaxation of the cricopharyngeus muscle, which was time-elapsed from the decrease in electromyogram of this muscle associated with swallow until electromyogram activity returned to its pre-swallow level. The amplitude measures were marked as the largest amplitude during the electromyogram burst. Electromyogram data were normalized (% of maximum) in each experiment to the maximum burst during swallowing, to enable comparison across animals.

A mean ± standard error was calculated for each animal including all swallows, and then averaged for each condition across animals. For statistical analysis an ANOVA with Fisher’s least significant difference post-hoc tests were performed on the amplitude and duration measures. Pearson’s product moment correlations (r) were calculated comparing all amplitude and duration measures. A difference was considered significant if the *P*-value was less or equal to 0.05.

## Results

**Figure 1:**
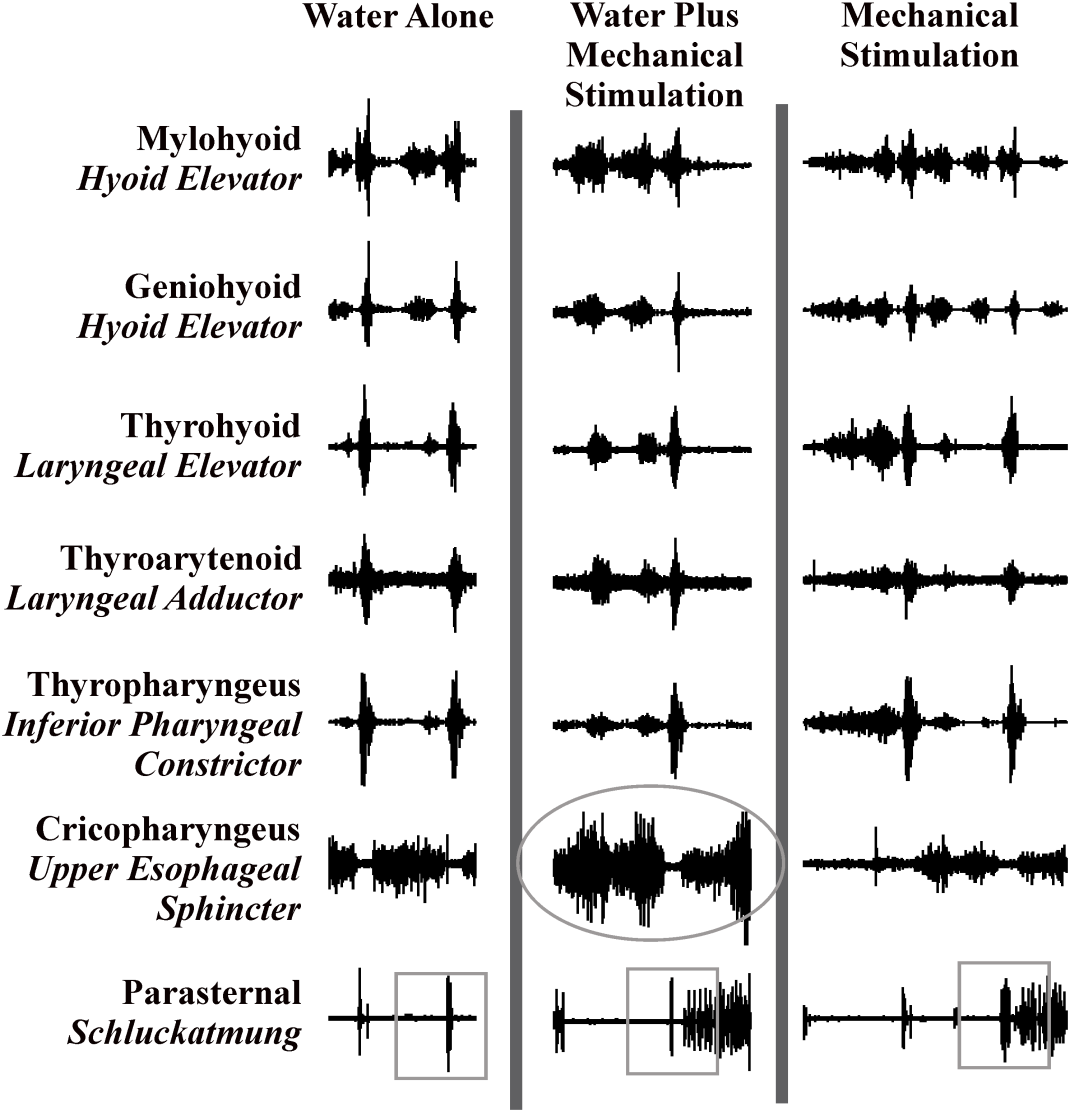
Raw electromyographic traces of swallow stimulated by the three conditions. Note the increased burst amplitude of the mylohyoid and post-swallow cricopharyngeus with the addition of water, and increased duration of the parasternal muscle electromyogram during pharyngeal swabbing.

We conducted 54 swallow trials in six animals. Figure 1 is an example of electromyogram motor responses across all three stimulus conditions. Each type of stimulus (water alone, water plus punctate stimulation, and pharyngeal swabbing) was effective in eliciting repetitive swallowing. Note that pharyngeal stimulation also elicited aspiration reflexes, which produce a sharp and sudden rise in inspiratory drive/pressure. During swallowing, laryngeal elevators, adductors, and pharyngeal constrictors all were activated in a ballistic-like manner (Figure 1) during each stimulus condition. Cricopharyngeus (upper esophageal sphincter) decreased activity during these ballistic-like bursts of other upper airway muscles (Figure 1). Table 1 summarizes electromyogram amplitude (percent of maximum) and duration (ms) mean for each muscle and condition.

**Table 1:** Effect of swallow stimuli on normalized electromyogram amplitude (% of maximum) and durations (ms) of selected swallow-related muscles, over the three stimulus conditions.

	Water	Water +	Pharyngeal
Amplitude (% max)	Only	Punctate Stimulation	Swabbing
***Hyoid/Laryngeal Elevators*** Mylohyoid	56±3	55±5	45±6*
Geniohyoid	59±8	58±8	52±9
Thyrohyoid	71±3	71+5	71+7
***Laryngeal Adductor*** Thyroarytenoid	70±5	63±5	63±6
***Pharyngeal*** Thyropharyngeus	72±4	69±4	68±4
Cricopharyngeus *Post-burst*	70±5*	57±6	40±6
***Schluckatmung*** Parasternal	41±7	41±9	57±7*
	**Water**	**Water +**	**Pharyngeal**
**Duration (ms)**	**Only**	**Punctate Stimulation**	**Swabbing**
***Hyoid/Laryngeal Elevators*** Mylohyoid	310±28	318±28	334±21
Geniohyoid	218±23	219±26	218±17
Thyrohyoid	243±28	248±37	286±32*
***Laryngeal Adductor*** Thyroarytenoid	308±60	320±53	378±46*
***Pharyngeal*** Thyropharyngeus	241±34	240±38	247±42
Cricopharyngeus-*Relaxation*	334±35	364±30	400±50
***Schluckatmung*** Parasternal	163±50	209±78	335±49*
***General***			
Laryngeal Elevation	319±28	316±27	327±20
Total Swallow Duration	333±33	386±50	321±32

*Significant effect (repeated measures ANOVA, P<0.05), significant difference (post-hoc test) from both other stimuli conditions (P<0.05).

### Electromyographic Amplitudes during Swallow

Stimulus condition had a significant effect for mylohyoid (hyoid elevator) amplitude, *F*(2,10) = 7.368, *P* = 0.01, and cricopharyngeus post-burst, *F*(2,10) = 15.243, *P* = 0.001. Electromyogram magnitudes of these muscles were significantly different during stimuli that included pharyngeal swabbing relative to water (mylohyoid *P* = 0.03; cricopharyngeus *P* = 0.004) and water with punctate stimulation (mylohyoid *P* = 0.001; cricopharyngeus *P* = 0.05) (Table 1). In addition, cricopharyngeus post swallow burst was significantly different during water relative to water with punctate stimulation (*P* = 0.005) (Table 1).

Stimulus condition had a significant effect for parasternal amplitude, *F*(2,10) = 7.435,*P* = 0.01. Unlike upper airway muscles, parasternal electromyographic magnitudes increased during pharyngeal swabbing relative to water alone (*P* = 0.001) and water with punctate stimulation (*P* = 0.03) (Table 1). No significant stimulus-related effects were observed for geniohyoid (*P* = 0.36) thyrohyoid (*P* = 0.98), thyroarytenoid (*P* = 0.33), and thyropharyngeus (*P* = 0.46).

### Electromyographic Burst Durations during Swallow

Stimulus condition had a significant effect for thyrohyoid electromyogram burst durations, *F*(2,10) = 4.093, *P* = 0.05. Thyrohyoid muscle electromyogram burst durations were significantly increased during pharyngeal swabbing relative to water with punctate mechanical stimulation (*P* = 0.05) (Table 1).

Stimulus condition also had significant effects for thyroarytenoid, *F*(2,10) = 5.249, *P* = 0.02, and parasternal, *F*(2,10) = 5.692, *P* = 0.02, muscle electromyogram burst durations. Electromyogram burst duration for both muscles were significantly longer for swallows induced by pharyngeal swabbing relative to water alone (thyroarytenoid *P* = 0.03; parasternal *P* = 0.006); however, magnitude of increase in burst-duration for parasternal muscle was greater than that observed for thyroarytenoid during swallows elicited by pharyngeal swabbing (Table 1).

No significant stimulus-related effects were observed for mylohyoid (*P* = 0.37) geniohyoid (*P* = 0.99), thyropharyngeus (*P* = 0.93), cricopharyngeal relaxation (*P* = 0.16), laryngeal elevation (onset of mylohyoid to termination of geniohyoid burst) (*P* = 0.83), and total swallow duration (*P* = 0.12).

### Person Product Moment Correlations (*r*)

**Figure 2:**
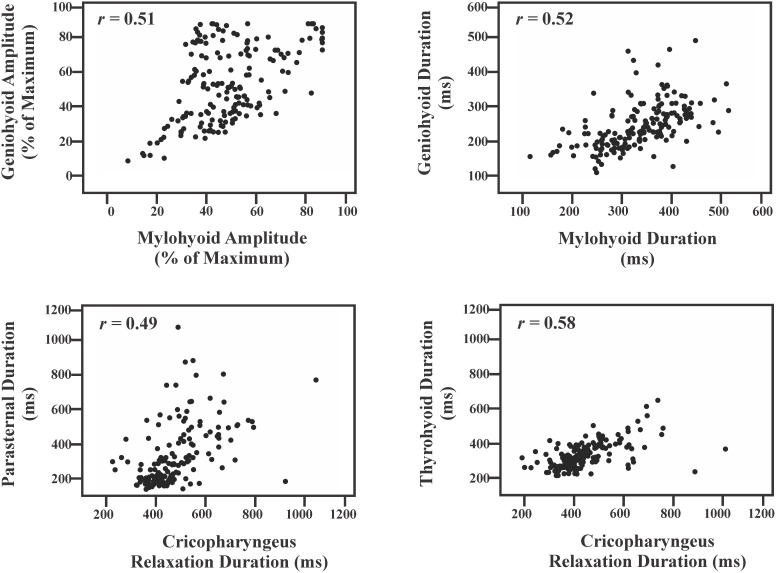
Pearson product moment correlation scatter plot examples for the comparisons with the largest *r* values.

A matrix showing all Pearson Product moment correlations is shown in Table 2 and examples of high correlations in Figure 2. Two of 21(9%) comparisons of all the maximum electromyogram amplitudes resulted in moderate-positive relationships: mylohyoid and geniohyoid (*r* = 0.51; *P*<0.01), and geniohyoid and thyrohyoid (*r* = 0.48; *P*<0.01). Three of 21 (14%) comparisons of maximum electromyogram amplitudes resulted in weak-positive relationship: mylohyoid and thyrohyoid (*r* = 0.34; *P*<0.01), mylohyoid and cricopharyngeus burst (*r* = 0.30; *P*<0.01), and geniohyoid and thyroarytenoid (*r* = 0.36; *P*<0.01).

**Table 2:** Pearson Correlations comparing electromyogram amplitude and duration during swallowing.

		Amplitude							Duration						
		MyHy	GeHy	ThHy	ThAr	ThPh	CrPh	PS	MyHy	GeHy	ThHy	ThAr	ThPh	CrPh	PS
**Amplitude**															
*Hyolarngeal Elevators*	MyHy	x	**0.5**	**0.3**	0.1	0.1	0.3	0.2	−0.1	−0.1	−0.1	0.0	−0.1	−0.1	−0.2
	GeHy	x	x	**0.5**	**0.4**	0.1	0.1	0.0	0.2	0.1	0.2	0.0	0.2	0.1	0.1
	ThHy	x	x	x	0.2	0.2	0.0	−0.2	0.1	0.0	0.0	0.1	−0.1	0.0	0.1
*Laryngeal Adductor*	ThAr	x	x	x	x	0.3	0.1	−0.2	0.1	0.0	0.0	0.0	0.2	−0.2	0.0
*Pharyngeal*	ThPh	x	x	x	x	x	0.3	−0.3	0.0	−0.1	−0.1	0.0	0.0	−0.1	0.0
	CrPh	x	x	x	x	x	x	−0.1	−0.2	−0.1	−**0.4**	0.0	−0.2	−0.3	−0.3
*Schluckatmung*	PS	x	x	x	x	x	x	x	0.0	0.1	0.1	0.1	0.0	0.0	−0.2
**Duration**															
*Hyolarngeal Elevators*	MyHy	x	x	x	x	x	x	x	x	**0.5**	**0.6**	0.3	0.3	**0.4**	**0.4**
	GeHy	x	x	x	x	x	x	x	x	x	**0.7**	**0.5**	**0.5**	**0.4**	**0.3**
	ThHy	x	x	x	x	x	x	x	x	x	x	**0.4**	**0.4**	**0.6**	**0.5**
*Laryngeal Adductor*	ThAr	x	x	x	x	x	x	x	x	x	x	x	0.2	0.2	0.3
*Pharyngeal*	ThPh	x	x	x	x	x	x	x	x	x	x	x	x	0.2	0.0
	CrPh	x	x	x	x	x	x	x	x	x	x	x	x	x	**0.5**
*Schluckatmung*	PS	x	x	x	x	x	x	x	x	x	x	x	x	x	x
**Bold**- an *r* value above 0.30 (or below −0.3).															

All data was pooled over the three conditions: water only, water plus punctate mechanical stimulation and pharyngeal swabbing.

One of 21 (5%) comparisons of electromyogram durations resulted in a strong relationship: geniohyoid and thyrohyoid (*r* = 0.70; *P*<0.01). Eleven of 21 (52%) duration comparisons resulted in moderate relationships: mylohyoid and geniohyoid (*r* = 0.53; *P*<0.01), mylohyoid and relaxation of cricopharyngeus (*r* = 0.40; *P*<0.01), mylohyoid and parasternal (*r* = 0.40; *P*<0.01), geniohyoid and thyroarytenoid (*r* = 0.51; *P*<0.01), geniohyoid and thyropharyngeus (*r* = 0.53; *P*<0.01), geniohyoid and relaxation of cricopharyngeus (*r* = 0.43; *P*<0.01), thyrohyoid and thyropharyngeus (*r* = .40; *P*<0.01), thyrohyoid and relaxation of cricopharyngeus (*r* = 0.58; *P*<0.01), thyrohyoid and parasternal (*r* = 0.50; *P*<0.01), and relaxation of cricopharyngeus and parasternal (*r* = 0.49; *P*<0.01). Two of 21 (9%) duration comparisons resulted in weak-positive relationships: geniohyoid and parasternal (*r* = 0.31; *P*<0.01), and thyrohyoid and thyroarytenoid (*r* = 0.36; *P*<0.01).

None of amplitude versus duration comparisons resulted in significant-positive relationships with an exception of negative correlation between cricopharyngeus amplitude and duration of thyrohyoid (*r* = −0.38; *P*<0.01).

## Discussion

The results demonstrated differences in the pattern of pharyngeal muscles activation during pharyngeal swallow depending on the stimulus initiating the behavior. Pharyngeal swabbing resulted in a decrease of the post-swallow cricopharyngeus maximum activity (upper esophageal sphincter) and a significant increase in parasternal (schluckatmung) maximum activity, along with an increase burst duration in the thyrohyoid (laryngeal elevator), thyroarytenoid (laryngeal adductor) and parasternal. Additionally, Pearson correlations showed predictable positive linear relationships in muscles with have similar mechanical action (Table 2, Figure 2) however, no positive significant correlations between burst duration versus electromyogram amplitudes.

Mechanical stimulation of the pharynx, in particular pharyngeal swabbing, can differentially activate pharyngeal afferents, e.g. activation of mechanosensory receptors that do not respond to a stream of air, a jet of air, and/or those responding to chemical stimulation [46]. It is known in rats that mechanical stimulation of the pharynx elicits swallow mediated by sensory pathway through the pharyngeal branch of the glossopharyngeal nerve [47] Differentiate sensory information inducing swallow (pharyngeal swabbing vs. water) and/or a feedback from an execution of swallows with and without water may account for differences in activation of muscles we observed for stimuli applied (Table 1). In our experiments, pharyngeal swabbing with a cotton swab reliably elicited swallows and also another reflex behavior aspiration reflex. Aspiration reflex is a sudden and rapid rise in inspiratory drive (sniff-like) which has been frequently demonstrated in cats from mechanical stimulation of the pharynx [48]–[50], and in this study was not present in the other stimulus conditions. Aspiration reflex was well differentiated from swallow and alterations muscles activity due solely to the presence of aspiration reflex is unlikely. Doty and Bosma [2] also reported this behavior, but had difficulty discerning between aspiration reflex and swallow because of the limited electromyograms (only geniohyoid and thyrohyoid) that they recorded during their stimulus condition.

The pharyngeal swabbing had specific effect on the amplitude and duration of the parasternal activity (schluckatmung). Schluckatmung is a German word for “swallow breath” or the inspiratory (phrenic) drive during swallowing [51]–[55]. Increased inspiratory motor activity during swallowing has been reported in cats [56], goats [42], and humans (adult and infant) [39], [57]. McConnell has also published a series of papers describing this as the “hypopharyngeal suction pump” [58]–[72], and the first negative deflection in studies using esophageal pressure to describe swallow [55], [73], [74]. This is the first report of alterations of schluckatmung, the inspiratory drive during swallowing, as a result of varying the swallow stimuli. The role of this inspiratory activity in the coordination of breathing/swallowing and the bio-mechanics of bolus movement has not been fully established.

Injection of water into the pharynx is a reliable initiator of swallow [5], [6], [10], [45]. Even with removal of the whole soft palate and epiglottis, pharyngeal wall stimulation will elicit swallow[45]. Single sensory fibers of the superior laryngeal nerve innervating the pharynx consistently discharge to a water stimulus [6]. In the larynx the properties of water can alter the reflex response [6], [75]. Storey [10], [76] and Miller and Sherrington [45] demonstrated that the properties of water injected into the mouth (osmotic or trace calcium), milk, or “meat-juice” did not alter swallowing. However, sucrose, quinine-HCl, acetic acid, and ethanol applied to the pharynx evoked more swallows than water alone [75]. Ours is the first study to demonstrate that the pattern of the pharyngeal phase of swallow may be significantly altered depending on the swallow inducing stimulus. One marked difference between solely mechanical stimuli and water is the additive effect of a bolus moving through the pharynx. Miller [6] discussed the effect of static versus dynamic stimulus types, and the alteration of the patterned sensory input which would vary as the bolus crosses various receptive fiends moving through the pharynx (feedback sensory information). The water bolus also passes over and would activate sensory fields of the epiglottis, which can activate the apneic reflex and occasionally swallowing [45], [77]. Much of the previous work on swallowing quantified this behavior solely by number of occurrences in response to each stimulus or the delay from the onset of the stimulation to the initiation of the swallow. Our data have shown that analysis of swallow as a “digital” event significantly underestimates the complexity of this behavior. The spatiotemporal features of swallow are routinely evaluated during clinical fluoroscopy and this information is a critical determinant in the diagnosis and treatment of dysphagia [27], [78]–[80].

Finally, this is the first report of positive relationships between upper airway muscle electromyogram burst durations and amplitudes during swallowing (Table 2). Several of the more robust relationships could have been predicted based on their function during swallow, i.e. mylohyoid and geniohyoid burst duration and amplitude which work in concert to raise the hyolaryngeal complex during swallowing. Additionally, the cricopharyngeus and thyrohyoid muscles work in concert to open the upper esophageal sphincter and the strong positive linear relationship between the burst durations of the two muscles during swallow is consistent with their linked function. However, besides negative correlation between the amplitude of cricopharyngeus and the duration of thyrohyoid activation, there were no significant relationships between amplitude and duration measures for any of the muscles. This suggests differential mechanism for amplitude and electromyogram burst duration during swallow, which is similar to what has previously been found with cough, another airway protective behavior. Wang and colleagues [29] showed in anesthetized cats that there was no relationship between cough inspiratory or expiratory amplitude and the duration. Both behaviors (cough and swallow) are hypothesized to be controlled by brainstem networks of neurons which participate in the regulation of breathing [81]–[84]. These networks drive oral, pharyngeal, laryngeal, chest wall, and abdominal muscles. The nucleus tractus solitarius has been hypothesized as one site gating cough Bolser [85]. Furthermore, this gating mechanism may be separated into second-order neuron circuits which control different portions of the cough pattern, for example inspiratory, expiratory and the compression phase through laryngeal motor control. For swallow, Ootani and colleagues [86] also demonstrated convergence of afferents from the superior laryngeal nerve and the glossopharyngeal nerve for 80% of neurons in the nucleus tractus solitarius. The authors hypothesized that this convergence allows for integration or gating of information and is not just a substrate for a sensory-relay for swallow. The absence of correlation between phase duration and burst amplitude during swallow may be due in part to the gating or segregation of processing of afferent information in the nucleus tractus solitarius.

## Conclusion

Our findings prove the hypotheses that the spatiotemporal control of pharyngeal muscles involved in hyoid elevation and pharyngeal constriction would be modifiable based on stimulus modality. Additionally, the results support separate regulatory mechanisms for phase durations and amplitudes of related muscles activities during swallow. Often in clinical trials of dysphagia duration is a major outcome and is used to infer force production, in light of these results the use of duration may significantly limit its ability to predict force production. Measurements separately evaluating muscle amplitude/force and swallow duration should be considered when developing therapeutic interventions to rehabilitate dysphagia.
